# Defining and modeling dynamic spatial heterogeneity within tumor microenvironments

**DOI:** 10.1016/j.ceb.2024.102422

**Published:** 2024-10

**Authors:** Bethany Bareham, Matthew Dibble, Maddy Parsons

**Affiliations:** Randall Centre for Cell and Molecular Biophysics, King's College London, New Hunt's House, Guy's Campus, London, SE1 1UL, UK

## Abstract

Many solid tumors exhibit significant genetic, cellular, and biophysical heterogeneity which dynamically evolves during disease progression and after treatment. This constant flux in cell composition, phenotype, spatial relationships, and tissue properties poses significant challenges in accurately diagnosing and treating patients. Much of the complexity lies in unraveling the molecular changes in different tumor compartments, how they influence one another in space and time and where vulnerabilities exist that might be appropriate to target therapeutically. Recent advances in spatial profiling tools and technologies are enabling new insight into the underlying biology of complex tumors, creating a greater understanding of the intricate relationship between cell types, states, and the microenvironment. Here we reflect on some recent discoveries in this area, where the key knowledge and technology gaps lie, and the advancements in spatial measurements and in vitro models for the study of spatial intratumoral heterogeneity.

## Introduction

Solid tumors exhibit extensive heterogeneity both within tumor microenvironments (TME) of the same individual and among patients with the same disease type [1]. This heterogeneity complicates clinical diagnosis and treatment, leading to poorer patient outcomes [1,2]. Incorporating spatial heterogeneity into prognosis scores outperforms marker expression averages as a prognostic factor in colorectal cancer [2]. While genetic and epigenetic changes are critical in cancer initiation, the spatial relationships between cancer cells and TME components, such as stromal cells and the extracellular matrix (ECM), are equally important in disease progression and treatment response. This is true even for tumors with common genetic drivers [3]. Spatial heterogeneity is a feature of many solid cancers, with distinct signatures at the leading edge (LE) versus the tumor core (TC), indicative of transition states to invasive disease. Dynamic changes to the mechanical microenvironment also influence cell behavior, signaling, tumor evolution, and heterogeneity [4], but these ‘unseen’ physical properties remain challenging to measure at high resolution and to interpret in context. Understanding the impact of these combined dynamic spatial changes on cancer evolution holds great promise as a means to identify and target drug resistance features and pathways at an early stage [5,6]. Here we focus on recent insights into mechanical, cellular, and signaling heterogeneity in the LE compartment using spatial profiling approaches and models of human disease. Given the importance of the LE in the transition to invasive disease, we aim to draw out emerging spatial relationships between the LE and TME that relate to heterogeneity in terms of treatment response. Emerging findings shed light on the fundamental biology of spatially heterogeneous and hard-to-treat cancers and offer new opportunities for personalized biomarker development and therapeutic options.

## Spatially distinct mechanical signatures in the tumor microenvironment

**Figure 1:**
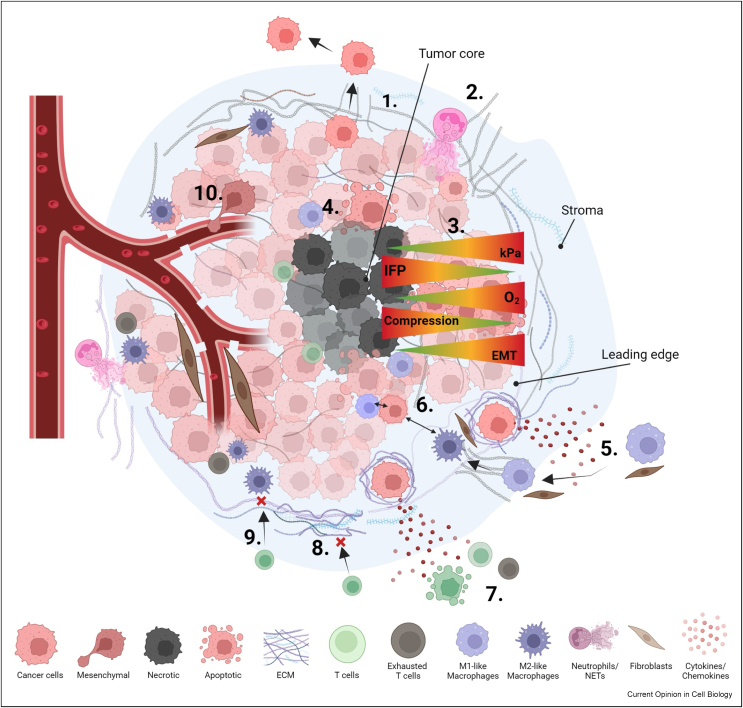
**Spatial heterogeneity within the tumor microenvironment.** Schematic illustrating examples of distinct immune and biomechanical processes occurring at the leading edge (LE) and core (TC) of solid tumors. **1.** Deposition of collagen I occurs at the LE, linearly arranged and perpendicular to the tumor mass aiding in cancer cell invasion. **2.** ECM is deposited by various cells, including cancer cells and cancer-associated fibroblasts (CAFs). Neutrophils also deposit neutrophil extracellular traps (NETs) which interact with ECM proteins, promoting local tumor cell invasion. **3.** When compared to the TC, the LE has increased stiffness and epithelial-to-mesenchymal transition (EMT). Whereas the TC has increased hypoxia, interstitial fluid pressure (IFP), and compression. **4.** This increased hypoxia and solid stress can lead to tumor cell apoptosis, driving the formation of a necrotic core. **5.** Tumor cells within the LE recruit macrophages and CAFs to the tumor site via chemotaxis, where tumor-associated macrophages (TAMs) are polarized towards an M2 phenotype expressing CD68, CD206, and CD163 markers. **6.** TAM phenotype is influenced by proximity to cancer cells, with high proximity correlating with CD68+ IRF8+ M1-like TAMs. **7.** Collagen IV primes cancer cells to release more CXCL10. CXCL13 and CCL2 cytokines resulting in increased Th1 differentiation, T cell apoptosis, and T cell exhaustion. **8.** The dense ECM at the LE acts as a barrier for infiltrating T cells. **9.** TAMs also act as a barrier, preventing the infiltration of T cells. **10.** EMT at the LE promotes metastasis as mesenchymal-like cancer cells intravasate into blood vessels and extravasate to secondary sites. Immature, leaky vasculature is present throughout the TME and contributes to the increased IFP observed.

Cells within LE and TC zones respond to distinct localized mechanical and chemical cues, resulting in segregated phenotypes and cell-type organization across the tumor (Figure 1). Medical imaging approaches such as MR- and ultrasound-based elastography have identified altered viscoelastic properties in malignant lesions compared to healthy tissue, most notably in breast, liver, brain, and pancreas [7]. However, the low resolution of these images limits the understanding of cell-level heterogeneity. Atomic Force Microscopy (AFM) applied to patient biopsies reveals stiffnesses ranging from 1.13 to 1.83 kPa in healthy breast tissue, 1.91 to 3.68 kPa in benign lesions, and 0.31 to 20 kPa in breast cancer [8]. Higher stiffness correlates with loss of mammary gland architecture, breast tumor vascularization, and increased tumor-adjacent ECM [8, 9, 10]. The 2-dimensional nature of AFM limits the analysis of precise biomechanical heterogeneity and cell-type relationships within the three-dimensional (3D) depth of solid tumors. Orthogonal approaches have shown the ECM adjacent to the LE exhibit thicker, more aligned collagen fibers in patients with poor prognosis vs. disease-free survival, which may partially explain the increased stiffness [11]. This is perpetuated by upregulation of the collagen cross-linking enzyme lysyl oxidase-like 3 (LOXL3) at the LE of invasive ductal breast carcinoma, which promotes local ECM stiffening and collective cancer cell invasion in 3D spheroid models [12]. Local transforming growth factor β (TGFβ) signaling can further drive mechanically induced transcription through mediators including the widely studied Yes-associated protein (YAP) and WW domain-containing transcription regulator protein 1 (TAZ), maintaining stiffness-induced spatial heterogeneity within the tumor compartment [10,13,14]. The machine-learning-based Spatially Transformed Inferential Force Map (STIFMap) tool predicts breast cancer stiffness using AFM biomechanical and collagen organization training datasets. This has revealed mechanical heterogeneity associated with markers of invasion and distinct epithelial mesenchymal transition (EMT) transcriptional mediators including ZEB1, suggestive of stiffness-induced transitions in LE cells [15]. Development of further AI-based tools to predict mechanical features that are coupled with regional cell-type classifiers and structural features would enable a more holistic understanding of how local tissue biophysics links to LE signatures in a range of cancer types.

## Leading edge features within heterogeneous tumors

Single-cell transcriptomics has revolutionized our ability to profile distinct heterogeneous populations from bulk tissue samples. However, it lacks spatial information critical for understanding the relationships between these cells and the TME. Spatial transcriptomics (ST) platforms provide powerful means to deep dive into the molecular pathways that contribute to regional heterogeneity in tumors and reveal common signatures across multiple tumor types (Figure 1). Notably, tumor biopsies used to characterize spatial heterogeneity typically represent a very small portion of the total TME, likely missing key features, and are vulnerable to sampling bias and variability due to the biopsy technique. Sampling errors can be mitigated by taking multiple biopsies across different regions and the characterization of larger biopsies.

The LE has distinct mechanical [8, 9, 10], structural [11], biochemical [16, 17, ∗18], enzymatic [12], signaling [10,13,14,∗19, 20, ∗21, 22], transcriptomic [∗19, 20, ∗21, 22, 23, ∗24], cellular [20,25], and immune [20,26, 27, 28, 29, ∗30, 31, 32, 33] properties. Emerging evidence also supports the notion that tumor cell states are inherently plastic, yet spatial distribution is relatively stable [24], underlining the importance of spatial interrogation. ST in oral squamous cell carcinoma (OSCC) has revealed enriched EGF, Ephrin, and Notch signaling pathways in the TC, indicating dominant TC–TC interactions and retention of epithelial-like cell states. Conversely, the LE displays interaction signatures and spatial partitioning of cell-ECM pro-invasive signals [19]. Similar findings are observed in skin squamous cell carcinomas (sSCC), where LE shows tumor-specific keratinocyte niches with EMT transcriptional profiles and upregulation of cell-ECM adhesion molecules ITGB1, CD151, and FERMT1 [20]. Integration of single-cell RNA sequencing and multiplexed ion beam protein markers imaging further refine these cell states, revealing local relationships between tumor-specific keratinocytes, endothelial cells, and fibroblasts indicative of cancer-driven niche modulation to facilitate invasion [20]. This spatial segregation of LE is also evident in breast cancer [21,22] and glioblastoma (GBM) [23]. Common cell states including a predominant partial EMT (LAMC2/VIM positive) signature at the LE are emerging across multiple tumor types, with this subset of LE cells showing high proximity to fibroblasts and endothelial cells indicating potential crosstalk to drive a pro-metastatic niche [24]. Conversely, niches of tumor cells at the LE expressing interferon response gene modules are proximal to macrophages and T cells, indicating that spatially distinct subgroups of LE cells may influence – or be controlled by – the TME [24]. ST thus provides critical insights into the interactions of niches in the LE, revealing cell–cell crosstalk patterns. Whether the predominance of some niches over others correlates with clinical outcome across multiple tumor types remains to be seen, as does the functional relevance of this to therapeutic targeting. However, further refining and identifying the emergence of LE mechanisms in early-stage aggressive disease may help to identify new druggable targets.

## Spatial immune cell heterogeneity and relationship to tumor leading edge

Dynamic changes to the tumor immune microenvironment (TIME) result in heterogeneous niches and immune suppression. Intra-tumoral spatial heterogeneity of the TIME is characterized by immune cell location, distance between cells, distribution of immune regulators, and spatial patterns [34] (Figure 1). Multiplex immunofluorescence and spatial RNA sequencing have revealed distinct immune cell distributions within spatial niches of tumors, with highly heterogeneous TIMEs linked to sub-optimal responses to immune checkpoint inhibition [35, 36, 37]. While inter-tumoral heterogeneity across a range of solid tumors impacts treatment response and personalized medicine [38, 39, 40, 41, 42], our focus here on intra-tumoral relationships between the LE and immune compartment.

Immune cells are first introduced to the TME via the LE, where ST has revealed immune-suppressive niches proximal to the LE in sSCC [20], melanoma [26], and liver cancer [27]. In nasopharyngeal cancer, stromal leukocytes proximal to cancer cells exhibit elevated exhaustion markers compared to immune-rich cancer cell islets [28] suggesting LE-associated cells influence the local immune compartment through direct interactions or paracrine signaling. ECM density and stiffness at the LE also contribute to establishing spatial immune heterogeneity (Figure 1). The ECM can act as a physical barrier hindering T cell infiltration [29] and stiffer ECM can promote migration and M2 polarization of macrophages preventing tumor clearance [30]. ECM receptors enriched at the LE, including leukocyte-associated immunoglobulin-like receptor-1 (LAIR-1), mediate collagen-induced immune suppression and are upregulated in macrophages, correlating with poor survival [31,43]. Similarly, osteoclast-associated receptor (OSCAR) positively correlates with cancer progression, metastasis, M2 macrophage polarization, and T cell exhaustion [32,33], illustrating the collaboration between immune cells and local ECM in regulating tumor immunity at the LE.

Tumor-associated macrophages (TAMs) are prevalent within the TIME, showing both functional and spatial diversity. Both their phenotype and proximity to the LE correlate with clinical outcomes [37]. Macrophages and cancer associated fibroblasts (CAFs) are recruited to the LE via tumor-derived cytokines, chemokines, and inflammatory responses [44]. In gastric cancer [37], non-small cell lung carcinoma [45] and colorectal cancer [46], M2-like TAMs are specifically found at the LE. This spatial heterogeneity may be influenced by contact-induced changes and cytokine gradients. For instance, M1-like (CD68+ IRF8+) TAMs are closest to cancer cells, whereas M2-like (CD68+ CD163+ CD206+) TAMs are located further away [36,37]. Thus, the spatial location and functionality of TAMs is influenced by the local microenvironment and interaction with cancer cells. Further exploration of the LE-immune spatial dynamics and TME physical properties can identify common signatures across cancers and enhance strategies to boost anti-tumor immunity and improve immunotherapy efficacy.

## Modeling dynamic spatial heterogeneity within the TME

**Figure 2:**
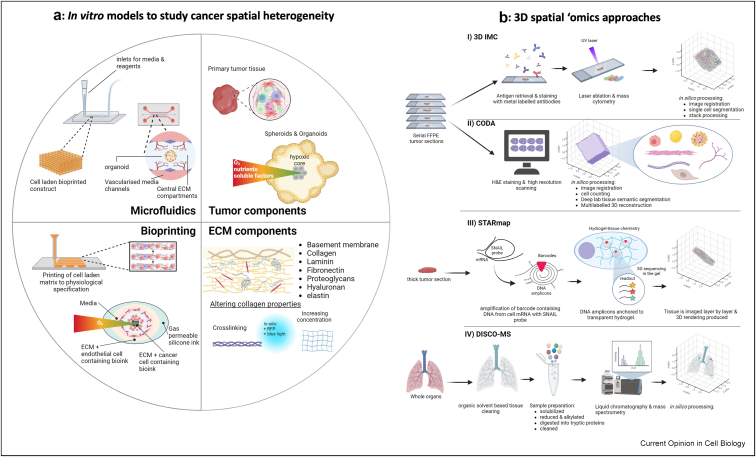
**Methodologies for the study of spatial heterogeneity** **(a) Modeling spatial heterogeneity in vitro**. **Tumor components:** tissue sections, organoids, and spheroids are commonly used in vitro to establish 3D tumor compartments. Ex vivo culture of tumor tissue samples retains a heterogenous population of cancer cells in addition to native TME components. Self-organized 3D spherical cell aggregates (spheroids) can be established from single or multiple cell lines. Organoids are established from tissue samples, iPSCs, ESCs, and ASCs through a process of enzymatic digestion and 3D culture with growth factors retaining a heterogenous population of cancer cells. Organoids and spheroids have gradient of nutrients, oxygen, and other soluble factors and often have a necrotic core. Other TME components that can be incorporated into 3D in vitro culture include immune cells, CAFs, ECM, vasculature, and secretory components. **ECM components:** ECM components can be sourced from decellularized ECM, artificial hydrogels, and isolated ECM-derived proteins (such as collagen, elastin, and hyaluronic acid). Stiffness can be regulated through crosslinking, increasing concentration of ECM components, or incubation of collagen with riboflavin phosphate (RFP) followed by blue light exposure. **Bioprinting:** layer-by-layer deposition of bioinks with predetermined and tunable ECM and cellular components for the creation of 3D scaffolds and TMEs. The example shows cancer cells seeded at the core with an ECM component and then surrounded by endothelial cells to establish components including TC, LE, and surrounding tissue. Over time an oxygen gradient forms, leading to hypoxia at the core. **Microfluidics:** integration of bioprinting and microfluidics allows for the introduction of flow-derived stress to recapitulate the dynamic TME. Microfluidic devices can also incorporate 3D cancer components such as organoids embedding them within an ECM and integrating with a vascular network to further mimic the TME and the LE-TC dynamics. **(b) 3D spatial ‘omics examples**. **I)** 3D Imaging mass cytometry (**3D-IMC**) in which serial tissue sections are stained with metal labeled antibodies. The surface of the sample is ionized turning molecules into ions allowing for the spatial detection and quantification of different ions. A 2D image is produced for each slide, images are then aligned and stacked to generate a 3D reconstruction [61]. **II) CODA** has been used to create 3D renderings of hematoxylin & eosin-stained tissue slides, serial images are aligned and stacked to create a 3D rendering, and machine learning is applied for the identification of several pathologically relevant TME compartments including cancer cells, epithelium, smooth muscle and nerves, fat, collagen, islets of Langerhans and acini [63]. **III)** Spatially resolved transcript amplicon readout mapping (**STARmap**) is a technique to create 3D renderings of thick tissue sections. Specific RNA molecules within the tissue are labeled with signal amplification by exchange reaction (SNAIL) probes. SNAIL probes facilitate amplification of RNA, during signal amplification unique fluorescent barcodes are introduced, specific to each target RNA. A hydrogel precursor solution is infused into the tissue and polymerized in situ to create a cross-linked hydrogel network that embeds the tissue. During polymerization functional groups within the hydrogel bind to DNA amplicons fixing them in place, ensuring they remain in their original spatial position. Tissue is cleared and imaged layer by layer to detect spatial location of fluorescent signals. 2D images are then aligned and compiled into a 3D map [62]. **IV)** 3D imaging of solvent-cleared organs (DISCO) by mass spectrometry **(DISCO-MS)**, whole tissue is fixed and cleared using the DISCO method and imaged. Regions of interest (ROI) are identified via deep learning algorithms. Ultra-high sensitivity mass spectrometry is used to perform proteomics at these ROIs, resulting in a spatially resolved proteome analysis of tissue within whole organs [68].

Emerging hypotheses from static spatial tissue data can be partly recapitulated using in vitro models, through manipulation of the TME and incorporation of tumor, immune, and other stromal compartments (Figure 2a). Inducing 3D collagen gel alignment perpendicular to the LE of breast cancer organoids enhances invasion, replicating observations in human tumors [12]. Similarly, in line with patient signatures, stiffening 3D matrices with non-enzymatic crosslinkers or increasing ECM concentration activates mechanosensitive signaling proteins YAP and ERK1/2 at the LE of breast cancer spheroids, driving a feed-forward pro-invasive spatial signaling niche [25,47]. Optical perturbation inducing collagen crosslinking or tension release at the LE provides additional means to study the effects of precise local changes of biomechanical properties on LE signaling dynamics [12,25,34]. Models incorporating stiffer 3D ECM and interstitial flow demonstrate biomechanical contributions to M2 polarization and anti-inflammatory cytokine release, enhancing macrophage and tumor cell migration [48,49]. Transmission of contractile forces from breast cancer cells in 3D collagen activates nearby macrophages, aiding their migration towards the LE [13]. Conversely, prolonged culture in high-density collagen matrix reduces cytotoxic T cell activity, upregulates regulatory T cell markers, and reduces proliferation compared to low-density matrix [50] reproducing TIMEs at the LE of several cancer types [19,29]. Collagen IV also primes cancer cells to upregulate immunomodulatory cytokines (CXCL10, CXCL3, CCL2), reducing T cell attachment, clearance of cancer cells, and increasing T cell apoptosis [51] as suggested from ST data [44]. These in vitro findings broadly recapitulate some common ST signatures showing the collagen-rich environment of the LE may induce T cell dysfunction and exhaustion, limiting immune infiltration and tumor clearance. Interestingly, tumor-adjacent nerves are emerging as potential regulators of the LE, as recently revealed in ST analysis of OSCC [52], and future models to incorporate this additional TME element will be important to explore functional crosstalk mechanisms.

While fully recapitulating all spatial properties in vitro remains challenging, emerging are enabling precise reproduction of complex spatial phenotypes observed in vivo. 3D bioprinting, depositing bio-inks layer-by-layer, is a versatile approach allowing for the spatial control of cellular [53], matrix [54], biochemical [55], and vascular [56] elements. For example, creation of multicellular bio-printed TME with a distinct TC surrounded by stromal cells enables increased ECM deposition over time [57]. Bioprinting can also be integrated into microfluidic devices to model increasingly complex biological processes observed within the TME. Spheroid cultures can incorporate additional cell types, including cancer, endothelial cells, and fibroblasts, allowing cell–cell and/or cell–ECM interactions at the LE to be probed over time [30]. Patient-derived organoids can reproduce some aspects of intra-tumor heterogeneity and hold promise for personalized approaches for immunotherapy, as recently demonstrated for melanoma [58]. Microfluidics can incorporate bioinks, spheroids, and organoids, embedded within 3D ECM and integrating with a vascular network to further mimic the TME and the LE-TC dynamics [59]. It remains to be seen whether LE-TC spatial heterogeneity features can be fully replicated in these models, but if achievable, they offer several benefits over mouse models in terms of speed and throughput for mechanistic interrogation and therapeutic testing [60].

## Taking spatial omics to the next dimension

Spatial multiomics technology is evolving rapidly, but how transcript and protein link to the chemical and physical microenvironment at single-cell resolution remains unknown. Given the importance of local lipid, metabolic, and elemental composition to protein turnover and organelle function, placing existing spatial data in context will be critical to fully understand and interpret identified cell state heterogeneity. Combining emerging high-resolution platforms to map spatial lipid species and metabolites [16], elemental [17], and proteome [18] coupled with pathological features offers exciting possibilities for future investigation, and to identify vulnerabilities for potential therapeutic intervention.

Processes within the TME are organized in 3D space, but spatial technologies largely provide 2D information, limiting a full understanding of cellular interactions and neighboring structures. Emerging technologies such as 3D imaging mass cytometry (IMC) [61], spatially resolved transcript amplicon readout mapping (STARmap) [62], multiplexed cyclic immunofluorescence, and CODA [63,64] are taking our understanding of LE-TC heterogeneity to another dimension (Figure 2b). 3D renderings of HER2+ ductal breast tumors have been created from serial IMC images of thick biopsies using a 3D watershed algorithm for voxel-level data analysis [61]. This revealed heterogeneous cell interaction patterns and proximity to vascular networks as well as subtle variations in immune cells and macrophage subtypes relative to the LE previously unseen in 2D samples [61]. CODA provides 3D reconstructions of serially sectioned PDAC tissue and machine learning-based identification of spatial compartments without the need for immunohistochemical labeling or mass spectrometry [63]. This approach identified invasive pancreatic cancer cells protruding from the central tumor along collagen fibers aligned to ductal, lobular, vascular, and neural structures of the pancreas, highlighting the importance of 3D ECM topography [63]. Tissue-clearing techniques combined with RNA/protein probes also enable visualization of TME components within large tissue volumes and whole organs at a single-cell level, including vasculature [65], immune system [66], and stroma [67], and can be combined with mass spectrometry to reveal protein signatures [68]. These advancements demonstrate how powerful spatial technologies can be leveraged and adapted to provide unique insights into LE, TME, and TIME 3D spatial heterogeneity.

## Conclusions and future perspectives

Our understanding of spatial heterogeneity in hard-to-treat solid cancers is advancing rapidly. This review touches on some of the key recent studies that have begun to define the full transcriptional landscape and niches in several prominent tumor types, but many more examples exist or are emerging. Powerful and innovative computational tools, including machine learning algorithms to dissect and model the local relationships between cells and with the TME will aid in the interpretation of these large and highly complex datasets combined with additional clinical metadata. It is highly likely however that transcriptional state alone will not be sufficient to explain the biological variation in these tissues. Combining ST with orthogonal or direct correlative imaging of the proteome, metabolome, lipidome, and genome will enable a more comprehensive understanding of shared and distinct spatial niches within heterogenous tumors. The question remains as to how these complex spatial niches can be fully replicated in vitro for mechanistic experimentation and drug development. Identifying the minimum components required to recapitulate specific spatial features will also be important to reduce the potential complexity of in vitro models. Given the strong influence of biomechanical features of tissues on the establishment of niches within the TME, any models should also consider – and ideally measure – those forces. Indeed, the dynamic evolution of these niches remains unclear, as does how therapies may change this already heterogeneous landscape. Better models can only be developed through understanding the underlying biology of human disease. The new frontier in spatial biology is delivering this at pace and holds significant promise to uncover novel targets to treat drug-resistant cancers.

## Declaration of competing interest

The authors declare that they have no competing interests.

## Data Availability

No data was used for the research described in the article.
